# Supplementary material for: Genomic and Metabolomic Insights into Secondary Metabolites of the Novel Bacillus halotolerans Hil4, an Endophyte with Promising Antagonistic Activity against Gray Mold and Plant Growth Promoting Potential
Source: Microorganisms. 2021 Dec 3;9(12):2508. doi: 10.3390/microorganisms9122508 (PMC8704346; doi:10.3390/microorganisms9122508)
Supplement: Supplementary file 1 [file microorganisms-09-02508-s001.zip › microorganisms-1474899-supplementary.pdf]

Table S1. Genes involved in plant growth promotion and colonization harbored in the *B. halotolerans* Hil4 genome.

Gene Name	Product	Function
ysnE	N-Acetyltransferase	IAA synthesis
trpA	Tryptophan synthase alpha chain	
trpB	Tryptophan synthase beta chain	
trpC	Indole-3-glycerol phosphate synthase	
trpD	Anthranilate phosphoribosyltransferase	
nos	Nitric oxide synthase oxygenase	Nitric oxide synthesis
alsS	Acetolactate synthase	2,3-butanediol and acetoin synthesis
alsD	Acetolactate decarboxylase	
ilvB	Acetolactate synthase large subunit	
ilvH	Acetolactate synthase small subunit	
bdhA	2, 3-butanediol dehydrogenase	
budC	Diacetyl reductase [(S)-acetoin forming]	
speA	Arginine decarboxylase	Spermidine synthesis
speB	Agmatinase	
speH	S-adenosylmethionine decarboxylase proenzyme	
speE	Polyamine aminopropyltransferase	
phy	3-Phytase	Phytase synthesis
phoR	Alkaline phosphatase synthesis sensor protein	Alkaline phosphatase
phoP	Alkaline phosphatase synthesis transcriptional regulatory protein	
phoA	Alkaline phosphatase 4	
phoD	Alkaline phosphatase D	
flgB	Flagellar basal body rod protein FlgB	Flagella biosynthesis
flgC	Flagellar basal-body rod protein FlgC	
flgK	Flagellar hook-associated protein 1	
flhA	Flagellar biosynthesis protein FlhA	
fliD	Flagellar hook-associated protein 2	
fliG	Flagellar motor switch protein FliG	
fliJ	Flagellar FliJ protein	
fliM	Flagellar motor switch protein FliM	
fliP	Flagellar biosynthetic protein FliP	
fliQ	Flagellar biosynthetic protein FliQ	
fliR	Flagellar biosynthetic protein FliR	
fliS	Flagellar secretion chaperone FliS	
cheA	Chemotaxis protein CheA	Chemotaxis
cheB	Protein-glutamate methylesterase	
cheD	Chemoreceptor glutamine deamidase CheD	
cheR	Chemotaxis protein methyltransferase	
cheY	Chemotaxis protein CheY	
cheW	Chemotaxis protein CheW	
swrC	Swarming motility protein SwrC	Swarming motility
bslB	Biofilm-surface layer protein B	Biofilm formation
bslA	Biofilm-surface layer protein A	
tasA	Major biofilm matrix component	

pgsA	Glycerol-3-phosphate 3-phosphatidyltransferase	Poly-glutamic acid (γ-PGA) synthesis
